# Enhanced Inflammation and Nitrosative Stress in the Saliva and Plasma of Patients with Plaque Psoriasis

**DOI:** 10.3390/jcm9030745

**Published:** 2020-03-10

**Authors:** Anna Skutnik-Radziszewska, Mateusz Maciejczyk, Iwona Flisiak, Julita Krahel, Urszula Kołodziej, Anna Kotowska-Rodziewicz, Anna Klimiuk, Anna Zalewska

**Affiliations:** 1 Experimental Dentistry Laboratory, Medical University of Bialystok, 1 Jana Kilinskiego Street, 15-089 Bialystok, Poland; anna.skutnik@o2.pl; 2 Department of Hygiene, Epidemiology and Ergonomics, Medical University of Bialystok, 2c Mickiewicza Street, 15-022 Bialystok, Poland; mat.maciejczyk@gmail.com; 3 Department of Dermatology and Venereology, Medical University of Bialystok, 14 Zurawia Street, 15-540 Bialystok, Poland; iwona.flisiak@umb.edu.pl (I.F.); julita.leonczuk@gmail.com (J.K.); 4 Department of Restorative Dentistry, Medical University of Bialystok, 24A M. Sklodowskiej-Curie Street, 15-276 Bialystok, Poland; ulakol@poczta.onet.pl (U.K.);; 5 Experimental Dentistry Laboratory, Medical University of Bialystok, 24A M. Sklodowskiej-Curie Street, 15-276 Bialystok, Poland; annak04@poczta.onet.pl

**Keywords:** plaque psoriasis, salivary glands, saliva, cytokines, nitrosative stress

## Abstract

Psoriasis is the most common inflammatory skin disease, characterized by the release of proinflammatory cytokines from lymphocytes, keratinocytes, and dendritic cells. Although psoriasis is considered an immune-mediated inflammatory disease, its effect on secretory activity of salivary glands and quantitative composition of saliva is still unknown. The aim of this study was to evaluate the secretion of saliva as well as several selected inflammation and nitrosative stress biomarkers in unstimulated and stimulated saliva as well as plasma of psoriasis patients. We demonstrated that, with progressing severity and duration of the disease, the secretory function of the parotid and submandibular salivary glands is lost, which is manifested as decreased unstimulated and stimulated saliva secretion and reduced salivary amylase activity and total protein concentration. The levels of tumor necrosis factor-alpha (TNF-α), interleukin-2 (IL-2), and interferon-gamma (INF-γ) were significantly higher, whereas interleukin-10 (IL-10) content was considerably lower in unstimulated and stimulated saliva of patients with psoriasis compared to the controls, and the changes increased with the disease duration. Similarly, we observed that the intensity of nitrosative stress in the salivary glands of psoriasis patients depended on the duration of the disease. By means of receiver operating characteristic (ROC) analysis, we showed that the evaluation of nitric oxide (NO), nitrotyrosine, and IL-2 concentration in non-stimulated saliva with high sensitivity and specificity differentiated psoriasis patients on the basis of the rate of saliva secretion (normal salivation vs. hyposalivation). In summary, the dysfunction of salivary glands in psoriasis patients is caused by inflammation and nitrosative stress.

## 1. Introduction

Psoriasis vulgaris is a skin inflammatory disease, the third most common among autoimmune diseases [1]. The pathogenesis of psoriasis involves the combination of genetic susceptibility, aberrant immune response, and several environmental factors (injuries, viral infections, medications taken, food intolerances). Typical features of psoriasis vulgaris include an immune-mediated process in which the key role is played by Th1 cells (T-helper 1). The presence of antigen-specific CD4+ (dendritic cell) T cells secreting type 1 cytokines: interferon-gamma (INF-γ), interleukin-2 (IL-2), and tumor necrosis factor-alpha (TNF-α) was observed in psoriatic skin lesions [2,3]. Moreover, the imbalance between Th1 and Th2 cells in psoriasis was confirmed by studies that showed interleukin-10 (IL-10) deficiency in psoriatic skin lesions [4]. Recent findings suggest that redox imbalance in the blood and skin of patients with psoriasis, resulting from the immune system stimulation, plays as important role in the pathogenesis of plaque psoriasis as the inflammatory process itself. It has been shown that, in the course of psoriasis, oxidative stress (OS) is primarily caused by reduced activity/concentration of antioxidants [5,6,7,8,9] and leads to increased oxidative modification of cellular elements of skin and plasma [6,10,11]. Plasma and erythrocyte product of lipid peroxidation (malondialdehyde, MDA) is considered a biomarker of plaque psoriasis exacerbation [12,13].

The detrimental inflammatory milieu and increased production of free radicals (ROS) associated with plaque psoriasis are not limited to the skin, but are also responsible for the growing number of comorbidities, including cardiological diseases, metabolic syndrome, chronic kidney disease, mood disorders, and salivary gland diseases [5,14,15,16,17].

Saliva produced by salivary glands performs numerous important functions in the oral cavity: hydrating it, removing harmful waste products and bacteria, participating in the remineralization of dental hard tissues, maintaining redox balance, and being involved in immune responses [18,19,20]. Disorders of both the composition and amount of saliva secreted into the oral cavity have physiological and psychological consequences. Therefore, it is very important to understand the mechanisms leading to salivary gland dysfunction in the course of systemic diseases. Unfortunately, the pathophysiology of salivary gland disorders in psoriasis is still unknown. In our previous work, we demonstrated that plaque psoriasis is accompanied by salivary redox imbalances with the prevalence of oxidation reactions. We observed that redox equilibrium in the submandibular glands was more vulnerable, and antioxidant capacity of the submandibular glands decreased with the disease duration [15]. There have been very few studies on the modification of saliva inflammatory components in psoriatic patients. Ganzetti et al. [21] demonstrated higher levels of TNF-α, transforming growth factor-beta (TGF-β), and interleukin-1 (IL-1β) in the saliva of psoriatic subjects vs. the controls. Unfortunately, most patients with psoriasis had also been diagnosed with periodontitis or gingivitis. Consequently, the observed cytokine changes in saliva reflected periodontal inflammation and not psoriasis-related salivary changes, and thus they did not explain the pathophysiology of salivary gland dysfunctions in the course of this disease. It has been evidenced that pro-inflammatory cytokines boost the expression of the inducible nitric oxide (NO) synthase (*i*NOS) in the cells, which results in increased NO synthesis in inflamed joints [22]. It was proven that NO and other reactive nitrogen intermediates affect a vast number of physiological functions of salivary glands, including exocytosis, water secretion, salivary blood flow, and non-specific immunological reactions [23,24,25]. The contribution of nitrosative stress to the pathophysiology of salivary glands in the course of psoriasis is unknown.

An ideal biomarker is characterized by simple determination as well as high sensitivity, specificity, and repeatability. It should also enable the identification of the patient’s physiological and pathological status and response to the applied treatment [26]. Numerous biomarkers have been suggested for easier diagnosis of psoriasis and monitoring of its treatment. However, studies on psoriasis biomarkers have been based on blood tests, examining skin fragments, conducting genetic tests, and transcriptomics. The results of these studies were divergent, and therefore neither was considered reliable nor was accepted as a psoriasis marker [27]. Saliva is a mixture of secretions of large salivary glands and gingival fluid. It also contains almost all the elements present in the blood and passing through the spaces between cells as part of an inter- and paracellular transport. It is also relatively easy and safe to collect, providing a new, non-invasive way to diagnose numerous general diseases [28,29,30,31,32].

Thus, the aim of this work was to explore the mechanisms responsible for salivary gland dysfunction in psoriasis. We compared the concentrations of TNF-α, IL-2, INF-γ, IL-10, NO, peroxynitrite, S-nitrosothiols, and nitrotyrosine in the saliva and blood of psoriatic subjects with hyposalivation and normal salivation vs. healthy controls. The goal of our study was also to search for salivary biomarkers to assess the severity of psoriasis and the accompanying salivary complications.

## 2. Materials and Methods

We obtained the consent of the Local Research Ethics Committee in Bialystok (permission number: R-I-002/563/2018). All patients as well as healthy subjects were informed about the purpose of the study and its risks and benefits, and they all consented to the collection of saliva and blood samples.

This experiment included psoriatic patients applying for treatment to the Department of Dermatology and Venereology of the Medical University of Bialystok. The reason for reporting to the hospital was exacerbation of psoriasis symptoms. Patients had not undergone general treatment of psoriasis within 2 preceding years. The only acceptable forms of treatment included local application of ointment with glucocorticosteroids, but not during 3 preceding months. The severity of skin lesions was assessed using the previously described Psoriasis Area and Severity Index (PASI) [15].

A total of 60 healthy patients participated in the study, who were individuals reporting for check-up visits to the Department of Restorative Dentistry at the Medical University of Bialystok, and were matched to the group of patients in terms of age and sex.

Within 6 preceding months, participants did not take any medicines that could affect the composition and secretion of saliva, as well as vitamins, antioxidants, and antibiotics. The participants from the control group were generally healthy, and patients with psoriasis did not have any accompanying diseases. The inclusion criteria was absence of periodontal pathology (periodontal pocket depth (PPD) < 2 mm, did not bleed during the probing) and no inflammatory or fungal changes on the oral mucosa. Only subjects without acrylic dentures were included in the study. The patients as well as healthy controls were not addicted to alcohol and did not smoke cigarettes.

One of the criteria differentiating the dysfunction of salivary glands is reduced flow of unstimulated saliva (NWS) [18]. Therefore, to delineate the influence of psoriasis vulgaris on the parotid and submandibular glands, psoriatic patients were divided into two groups: with reduced NWS (psoriasis hyposalivation-PH, *n* = 30) and normal NWS (psoriasis normal secretion- PN, *n* = 30). The limit value for NWS was assumed at ≤ 0.2 mL/min, which is considered the minimum value of NWS for healthy population [18]. Moreover, the minimum value of NWS in our control group was 0.21 mL/min.

The number of patients was confirmed by the test as sufficient, and the test power was 0.9. Clinical characteristics of the patients are presented in Table 1.

### 2.1. Blood Collection

Blood was collected at fasting, either at the admission of a patient to the hospital or during routine tests in case of control subjects. Blood was collected at 5 mL using an S-Monovette EDTA K3 tube (Sarstedt, Nümbrecht, Germany). The samples were then centrifuged (3000 × *g*, 10 min, 4 °C). No hemolysis was observed in any of the obtained plasma samples. To prevent sample oxidation, 0.5 M Butylated hydroxytoluene BHT (Sigma-Aldrich, Saint Louis, MO, USA; 10 µL/mL blood) was added [28]. Plasma was frozen (−82 °C). The samples were stored deep-frozen for no longer than 6 months.

### 2.2. Saliva Collection

The examined material was unstimulated and stimulated (SWS) total saliva collected from the patient by the spitting method [28]. Saliva was collected in the morning, on an empty stomach, between 8 a.m. and 10 a.m. in order to minimize the effect of daily changes on saliva secretion. The participants had refrained from taking any drugs for 8 hours prior to the examination. Saliva was collected in a separate room, in a sitting position with the head slightly inclined downwards, with minimized face and lip movements, and after a 5-minute adaptation period. Next, the patient rinsed the mouth three times with water at room temperature. The saliva collected during the first minute was discarded. The subsequent batches of saliva (the patient actively spat out the saliva accumulated in the bottom of the oral cavity) were collected into a plastic centrifuge tube placed in an ice container. NWS was collected for 15 minutes, and SWS was collected after a 5-minute break. Stimulation was performed by dropping 100 µL of 2% citric acid on the tip of the tongue every 20 seconds, for 5 minutes. To prevent sample oxidation, 0.5 M BHT (Sigma-Aldrich, Saint Louis, MO, USA; 10 µL/mL blood) was added to the saliva [33]. The volume of each sample was measured with a pipette calibrated to 0.1 mL. Saliva secretion was calculated by dividing the volume of the obtained saliva by the number of minutes of its collection. The collected saliva was centrifuged (20 minutes, 4 °C, 10,000 × *g*) [33]. The sediments were discarded, and supernatant fluid was divided into portions of 200 µL each, frozen in −80 °C, and stored until assayed, but for no longer than 6 months.

### 2.3. Stomatological Examination

After saliva collection, stomatological examinations were performed by one dentist (A.S.-R.), in artificial light, using a dental mirror, probe, and periodontal probe (Hossa International, Warsaw, Poland; design and construction in accordance with WHO guidelines) according to WHO criteria [34]. The dental status of each participant was assessed on the basis of the DMFT index (decayed, missing, filled teeth). The condition of periodontal tissues was assessed using GI (gingival index) and probing pocket depth (PPD) were assessed at teeth 16, 21, 24, 36, 41, and 44. The PPD and occurrence of bleeding were assessed after gently introducing the probe into the gingival space parallel to the long axis of the tooth, to the depth of perceived resistance posed by the bottom of the gingival gap. Four surfaces of each examined tooth (mesial, distal, buccal, and lingual) were examined. In the case of GI, we used a scale of 0 to 3. The sum of the values, from four surfaces divided by 4, determined the level of the PPD/ gingival index for a given tooth. Then, the results were added and divided by the number of teeth examined. In 25 participants, the inter-rater agreement between the examiner (A.S.-R.) and other experienced dentists (U.K., A.K.) was performed. The reliability for DMFT was *r* = 0.99, for GI was *r* = 0.89, and for PPD was *r* = 0.94. If bleeding during probing or PPD deeper than 2 mm were found, previously collected saliva was discarded and the patient was excluded from the study. Thus, 53 patients with psoriasis and 37 healthy controls who had bleeding of probing and/or PPD > 2 mm were disqualified from the study.

### 2.4. Biochemical Assays

All determinations of plasma, NWS, and SWS were performed in duplicate samples. The absorbance/fluorescence was measured using an Infinite M200 PRO Multimode Microplate Reader (Tecan Group Ltd., Männedorf, Switzerland). The results were standardized to 1 mg of protein.

### 2.5. Pro-Inflammatory Cytokines

The concentrations of TNF-α, IL-2, IL-10, and INF-γ were determined by the ELISA method using commercial kits from EIAab Science Inc. Wuhan (Wuhan, China), according to the manufacturer’s instructions.

### 2.6. Nitrosative Stress

The concentration of NO was determined spectrophotometrically using the Griess reagent—a solution of sulfanilic acid and α-naphthylamine in acetic acid [35]. The reaction of nitrates with sulfanilamide produces *N*-(1-naphthyl)-ethylenediamine dihydrochloride, the absorbance of which was measured at 490 nm wavelength.

The concentration of S-nitrosothiols was determined spectrophotometrically on the basis of the reaction of the Griess reagent with Cu^2+^ copper ions [36]. The solution was shaken and set aside for 20 minutes, and then the absorbance was measured at 490 nm [37].

Peroxynitrite concentration was determined spectrophotometrically via a test using peroxynitrite decomposition followed by nitration of 4-hydroxyphenylacetic acid (4-HPA) and glycyltyrosine [38]. The reaction resulted in the production of nitrophenol, the absorbance of which was measured at 320 nm wavelength.

The concentration of nitrotyrosine was determined by ELISA using the Nitrotyrosine ELISA kit from Immunodiagnostik AG (Bensheim, Germany), following the manufacturer’s instructions provided in the package.

### 2.7. Salivary Protein

Salivary protein content was determined using the bicinchoninic acid (BCA) method (Pierce BCA Protein Assay; Thermo Scientific (Rockford, IL, USA)). Bovine serum albumin was used as a standard.

### 2.8. Salivary Amylase

The activity of salivary amylase (EC 3.2.1.1.) was determined colorimetrically using 3,5-dinitrosalicylic acid (DNS) as a substrate [39]. By this method, starch was transformed by amylase to maltose, and was measured at 540 nm by the complex with DNS.

### 2.9. Statistical Analysis

The obtained results were assessed statistically by means of one-way analysis of variance (ANOVA). The significance of differences between individual groups was determined with the post-hoc Tukey’s HSD test, and normal distribution was confirmed via the Shapiro–Wilk test. The correlations between the examined parameters were described using the Pearson correlation coefficient. The value of *p* < 0.05 was considered statistically significant. In order to assess the diagnostic usefulness between plaque psoriasis patients with normal salivary secretion and hyposalivation, receiver operating characteristic (ROC) curves were generated and then the area under the curve (AUC) was calculated. Every parameter had its optimal limit values determined, which simultaneously provided high sensitivity and specificity. The analysis of the data was performed in the statistical program GraphPad Prism 8.3.0 for MacOS.

## 3. Results

### 3.1. Inflammatory Cytokines

#### 3.1.1. NWS

**Figure 1:**
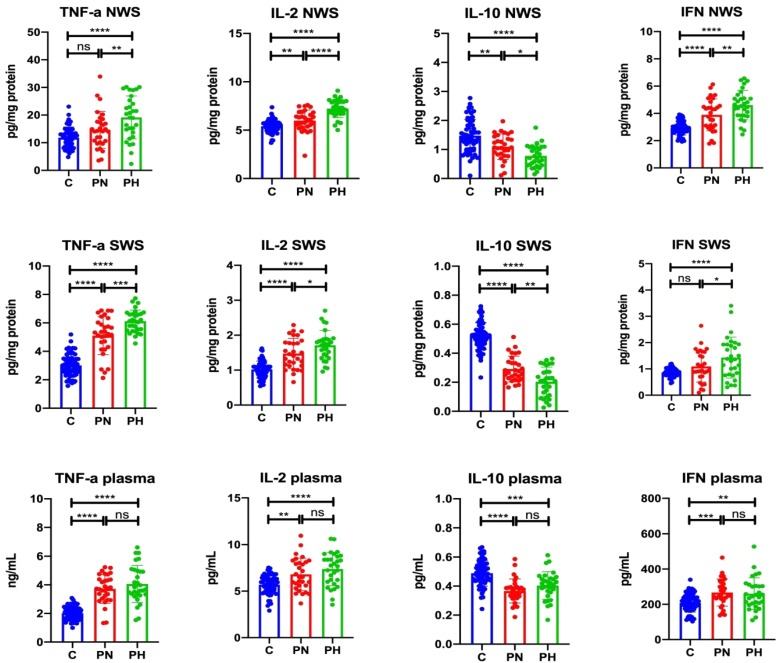
Cytokine levels in unstimulated and stimulated saliva as well as plasma of plaque psoriasis patients with normal salivation and hyposalivation. Abbreviations: C—the control; IL-2—interleukin 2; IL-10—interleukin-10; INF-γ—interferon-gamma; ns—not significant; NWS—non-stimulated whole saliva; PN—psoriasis patients with normal salivation; PH—psoriasis patients with hyposalivation; SWS—stimulated whole saliva; TNF-α—tumor necrosis factor-alpha. * *p* < 0.05, ** *p* < 0.01, *** *p* < 0.001, and **** *p* < 0.0001.

The concentration of IL-2 (↑10.91%, *p* = 0.007; ↑33.64%, *p* < 0.0001, respectively) and INF- γ (↑33.11%, *p* ≤ 0.0001; ↑57.34%, *p* ≤ 0.0001, respectively) in NWS of psoriasis patients with normal and decreased saliva secretion was significantly higher than in the control group. Moreover, concentrations of IL-2 (↑20.50%, *p* ≤ 0.0001) and INF-γ (↑18.21%, *p* = 0.005) in NWS of patients with hyposalivation were considerably higher than in psoriasis patients with normal saliva secretion. TNF-α concentration in the NWS of psoriasis patients with hyposalivation was significantly higher than in the control group (↑61.38%, *p* ≤ 0.0001) and in the group of psoriasis patients with normal salivation (↑30.47%, *p* = 0.009). The concentration of IL-10 (↓25.68%, *p* = 0.002; ↓47.30%, *p* ≤ 0.0001, respectively) in the NWS of psoriasis patients with normal and decreased saliva secretion was considerably lower than in the control group. Moreover, the concentration of IL-10 (↓29.09%, *p* = 0.03) in NWS of patients with hyposalivation was significantly lower than in the group of psoriasis patients with normal saliva secretion (Figure 1).

#### 3.1.2. SWS

TNF-α concentration (↑69.77%, *p* ≤ 0.0001; ↑103.32%, *p* ≤ 0.0001, respectively) and IL-2 (↑45.10%, *p* ≤ 0.0001; ↑67.65%, *p* ≤ 0.0001, respectively) in SWS of patients with psoriasis with normal and decreased saliva secretion was significantly higher than in the control group. The concentration of TNF-α (↑19.77%, *p* = 0.0002) and IL-2 (↑15.54%, *p* = 0.03) in SWS of patients with hyposalivation was considerably higher than in psoriasis patients with normal saliva secretion. INF-γ content in SWS of psoriasis patients with hyposalivation was significantly higher than in the control group (↑66.28%, *p* ≤ 0.0001) and in the group of psoriasis patients with normal saliva secretion (↑31.19%, *p* = 0.03). The level of IL-10 (↓44.23%, *p* ≤ 0.0001; ↓61.54%, *p* ≤ 0.0001, respectively) in SWS in psoriasis patients with normal and decreased saliva secretion was significantly lower than in the control group, with IL-10 content (↓31.23%, *p* ≤ 0.0001; ↓61.54%, *p* ≤ 0.0001, respectively) in SWS in hyposalivation patients significantly lower than in psoriasis patients with normal saliva secretion (Figure 1).

#### 3.1.3. Plasma

TNF-α concentration in plasma of psoriasis patients with normal secretion (↑83.25%, *p* ≤ 0.0001) and in plasma of psoriasis patients with hyposalivation (↑100.49%, *p* ≤ 0.0001) was significantly higher than in the control group. Similarly, plasma concentration of IL-2 and INF-γ in psoriasis patients with normal secretion (↑19.68%, *p* = 0.002; ↑28.51%, *p* = 0.0006, respectively) and hyposalivation (↑29.70%, *p* ≤ 0.0001; ↑25.29%, *p* = 0.003, respectively) were significantly higher vs. control. Plasma concentration of IL-10 in psoriasis patients with normal secretion (↓24.49%, *p* ≤ 0.0001) and hyposalivation (↓18.37%, *p* = 0.0001) was significantly lower than in the control group (Figure 1).

### 3.2. Nitrosative Stress

#### 3.2.1. NWS

The concentration of NO (↑14.31%, *p* = 0.009; ↑35.14%, *p* ≤ 0.0001, respectively) and nitrotyrosine (↑12.41%, *p* = 0.04; ↑39.60%, *p* ≤ 0.0001, respectively) in NWS of psoriasis patients with normal secretion and with hyposalivation was significantly higher than in the control group. Moreover, the levels of NO (↑18.23%, *p* = 0.0006) and nitrotyrosine (↑24.19%, *p* ≤ 0.0001) in NWS of patients with hyposalivation was considerably higher than in psoriasis patients with normal salivary secretion.

**Figure 2:**
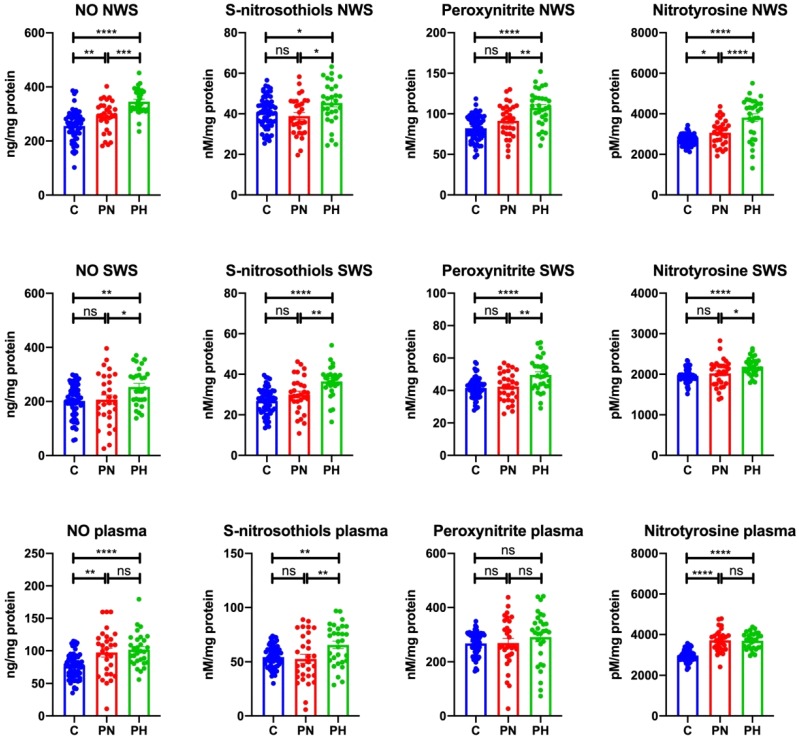
Nitrosative stress in non-stimulated and stimulated saliva as well as plasma of plaque psoriasis patients with normal salivation and hyposalivation. Abbreviations: C—the control; NO—nitric oxide; ns—not significant; NWS—non-stimulated whole saliva; PN—psoriasis patients with normal salivation; PH—psoriasis patients with hyposalivation; SWS—stimulated whole saliva. * *p* < 0.05, ** *p* < 0.01, *** *p* < 0.001, and **** *p* < 0.0001.

The concentration of S-nitrosothiols and peroxynitrite in NWS of psoriasis patients with hyposalivation was significantly higher than in the control group (↑11.59%, *p* = 0.04; ↑30.70%, *p* ≤ 0.0001, respectively) and the group of psoriasis patients with normal salivation (↑16.64%, *p* = 0.01; ↑17.63%, *p* = 0.003, respectively) (Figure 2).

#### 3.2.2. SWS

The concentration of NO, S-nitrosothiols, peroxynitrite, and nitrotyrosine in SWS of psoriasis patients with hyposalivation was significantly higher than in the control group (↑25.56%, *p* = 0.006; ↑35.93%, *p* ≤ 0.0001; ↑11.47%, *p* ≤ 0.0001; ↑11.80%, *p* ≤ 0.0001, respectively), as well as compared to the group of psoriasis patients with normal salivation (↑22.71%, *p* = 0.04; ↑21.53%, *p* = 0.002; ↑17.26%, *p* = 0.002; ↑8.26%, *p* = 0.02, respectively) (Figure 2).

#### 3.2.3. Plasma

The concentration of NO (↑27.19%, *p* = 0.001; ↑33.05%, *p* ≤ 0.0001, respectively) and nitrotyrosine (↑25.04%, *p* ≤ 0.0001; ↑24.34%, *p* ≤ 0.0001, respectively) in plasma of psoriasis patients with normal and decreased saliva secretion was considerably higher than in the control group. Plasma concentration of S-nitrosothiols in psoriasis patients with hyposalivation was significantly higher than in the control group (↑20.41%, *p* = 0.008) and in psoriasis patients with normal saliva secretion (↑24.18%, *p* = 0.008). Plasma concentration of peroxynitrite did not differ between the study and control groups (Figure 2).

### 3.3. Salivary Gland Function

**Figure 3:**
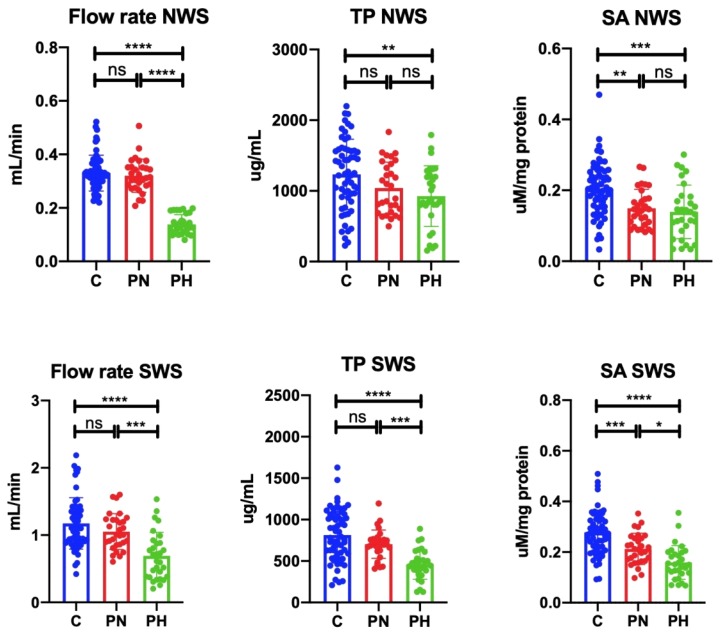
Salivary gland function in plaque psoriasis patients and control subjects. Abbreviations: C—the control; NWS—non-stimulated whole saliva; ns—not significant; PN—psoriasis patients with normal salivation; PH—psoriasis patients with hyposalivation; SA—salivary amylase; SWS—stimulated whole saliva; TP—total protein. * *p* < 0.05, ** *p* < 0.01, *** *p* < 0.001, and **** *p* < 0.0001.

Unstimulated as well as stimulated saliva secretion was significantly lower in psoriasis patients with hyposalivation compared to the control group (↓57.58%, *p* ≤ 0.0001; ↓41.03%, *p* ≤ 0.0001, respectively). Similarly, unstimulated as well as stimulated saliva secretion was significantly lower in psoriasis patients with hyposalivation compared to the psoriatic patients with normal salivation (↓56.25%, *p* ≤ 0.0001; ↓34.29%, *p* = 0.0003, respectively). The concentration of protein in NWS of psoriasis patients with hyposalivation was considerably lower than in the control group (↓24.90%, *p* = 0.008). Protein content in SWS of psoriasis patients with hyposalivation was significantly lower than in the controls (↓43.49%, *p* ≤ 0.0001) and the group of patients with normal saliva secretion (↓13.60%, *p* = 0.0008). The activity of salivary amylase in NWS of psoriasis patients with hyposalivation and normal salivation was visibly lower than in the control group (↓30.00%, *p* = 0.0003; ↓25.00%, *p* = 0.002, respectively). Similarly, salivary amylase activity in SWS of psoriasis patients with hyposalivation and normal salivation was significantly lower than in the control group (↓42.86%, *p* ≤ 0.0001; ↓25.00%, *p* = 0.0006, respectively). Moreover, amylase activity in SWS of patients with hyposalivation was considerably lower than in patients with normal salivation (↓23.81%, *p* = 0.02) (Figure 3).

### 3.4. ROC Analysis

**Figure 4:**
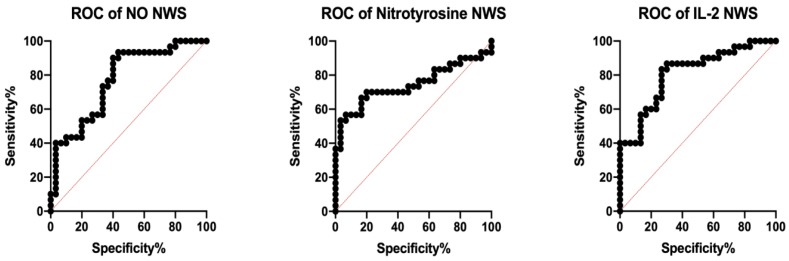
Receiver operating characteristic (ROC) analysis of nitric oxide, nitrotyrosine, and IL-2 in unstimulated saliva of plaque psoriasis patients with normal salivation and hyposalivation. IL-2—interleukin 2; NO—nitric oxide; NWS—non-stimulated whole saliva.

The assessment of diagnostic usefulness of the analyzed biomarkers of inflammation and nitrosative stress is presented in Table 2 and Table 3. Many of the assessed parameters clearly differentiated psoriatic patients with hyposalivation from patients with normal salivary flow. Particularly noteworthy is the assessment of NO, nitrotyrosine, and IL-2 levels in NWS, differentiating psoriatic patients with high sensitivity and specificity on the basis of the rate of saliva secretion (Figure 4).

### 3.5. Correlations

The results of statistically significant correlations are presented in Table 4. We demonstrated a negative correlation between NO concentration and minute secretion of unstimulated saliva as well as between peroxynitrite and protein concentrations in stimulated saliva of patients with hyposalivation. Moreover, we observed a negative correlation between TNF-α level and non-stimulated salivation, as well as in IL-2 content and stimulated salivary flow in patients with hyposalivation. On the other hand, peroxynitrite concentration correlated negatively with α-amylase activity in both unstimulated and stimulated saliva of patients with normal salivation.

We noted a positive correlation between TNF-α and NO concentrations in unstimulated saliva of patients with hyposalivation, as well as between IL-2 and NO contents in unstimulated saliva of psoriasis patients with normal saliva secretion.

We showed a positive correlation between PASI and TNF-α, as well as PASI and IL-2 in unstimulated saliva of patients with hyposalivation. Moreover, we observed a positive correlation between nitrotyrosine concentration and duration of psoriasis in patients with normal as well as reduced salivary flow (both in unstimulated and stimulated saliva).

## 4. Discussion

In the presented study, we evaluated the concentrations of TNF-α, IL-2, INF-γ, IL-10, and selected nitrosative stress parameters (NO, peroxynitrite, S-nitrosothiols, and nitrotyrosine) in NWS and SWS, as well as the plasma of psoriasis patients. The obtained results demonstrated the pathophysiology of salivary gland dysfunction in the course of plaque psoriasis. We were also seeking salivary psoriasis biomarkers that could be helpful in diagnosing the severity of psoriasis and its salivary complications.

The accepted values of normal unstimulated salivary flow are above 0.2 mL/min. Any unstimulated flow rate below 0.2 mL/min is considered salivary gland hypofunction and is referred to as hyposalivation [18]. Hyposalivation has a detrimental effect on numerous aspects of oral health, and thus on general well-being. It decreases the quality of life as it hinders speaking, tasting, chewing, and swallowing food [40]. Reduced saliva secretion is the cause of cracks and fissures in the oral mucosa, which is associated with chronic pain in the oral cavity and the resulting discomfort to the patient. Decreased salivation also contributes to boosted incidence of caries, periodontitis, and fungal infections of the oral cavity [40]. All these may lead to patient malnutrition, social isolation, and even depression, as well as generate high treatment costs. Therefore, it is very important to identify patients with salivation disorders and to prevent the development and effects of salivary gland dysfunction in the course of systemic diseases.

Our findings that plasma concentrations of TNF-α, IL-2, and INF- γ were significantly higher and for IL-10 were significantly lower than in the controls are consistent with the assumption that psoriasis is primarily driven by an aberrant immune response and results from an imbalance between Th1 and Th2 cells [2,3,4]. Significantly higher concentration of NO, especially nitrotyrosine, and a positive correlation between the latter and the disease duration in psoriatic patients’ plasma compared to the controls confirm the contribution of nitrosative stress to the development of the disease [41]. Interestingly, apart from S-nitrosothiols, we did not observe significant differences between patients with hyposalivation and those with normal saliva secretion. On the other hand, patients from the hyposalivation group were characterized by longer duration of psoriasis and higher PASI index compared to those with normal flow of unstimulated saliva.

At this point, it is worth reminding that 90% of saliva is produced by three pairs of large salivary glands: submandibular, parotid, and sublingual. The remaining 10% of saliva is secreted by small salivary glands scattered under the oral cavity mucosa, and gingiva fluid. The submandibular glands are the major contributor to unstimulated salivary flow, and the parotid glands secrete stimulated saliva, that is, saliva secreted mainly in response to stimuli. The contribution of the sublingual glands to unstimulated and stimulated salivation is low [42]. Therefore, any deviation in the composition of unstimulated saliva reflects dysfunction of the submandibular glands, as well as of stimulated saliva, of the parotid glands. The exception here are patients with inflammatory changes in periodontal tissues, in whom changes in saliva composition reflect periodontal diseases. In our work, we excluded patients with periodontitis/gingivitis, and therefore any changes observed in saliva originated from the dysfunction of salivary glands.

Our results revealed significantly increased levels of the tested proinflammatory cytokines and a decrease in IL-10 concentration in unstimulated and stimulated saliva of psoriasis patients with normal salivation (except for TNF-α in NWS and INF-γ in SWS) and hyposalivation compared to the controls. An earlier report suggested that mRNA expression of Th1 and derived inflammatory cytokines IL-2, TNF-α, and INF-γ were also increased in the saliva of the patients with Sjögren’s syndrome [43]. The salivary changes were accompanied by clusters of infiltrating cells present in salivary gland biopsy, where 80% were Th1 cells, and the remaining 20% consisted of stimulated B lymphocytes and plasma cells [43]. On the basis of the performed analyses, it was difficult to assess the nature of the developing inflammation in salivary glands of our patients. There were also no histological examinations of the salivary glands of psoriasis patients. By analogy to Sjögren’s syndrome, the observed increases in TNF-α, IL-2, and INF-γ concentrations allow us to assume that salivary glands of patients with psoriasis are infested with autoreactive Th1 lymphocytes. Despite the deficiency of Th2 response (↓IL-10) supporting the humoral type response, we do not rule out the presence of stimulated B lymphocytes, as there has been no research to confirm or exclude the existence of the culprit autoantigens. However, increased concentration of the examined proinflammatory cytokines and decreased level of IL-10 in NWS and SWS of patients with hyposalivation compared to psoriasis patients with normal salivation indicates an increase in imbalance between Th1 and Th2 cells, and thus inflammation in salivary glands of patients with hyposalivation vs. those with normal salivary flow.

Human salivary glands contain different kinds of nitric oxide synthase (NOS) isoforms. Neuronal NOS (*n*NOS) was found in the salivary gland parenchyma, ducts, blood vessels, and nerve fibers around acini, mainly in the submandibular glands [44], and—in negligible amounts—in the parotid and sublingual glands [45]. Endothelial NOS (*e*NOS) was identified as localizing to the glandular vascular endothelium of the salivary ducts [45]. *i*NOS has been detected in the salivary ducts of normal tissue [45]. In physiological concentrations, NO does not damage the structures of salivary glands; it regulates oral blood flow and saliva secretion, and participates in non-specific protective mechanisms [23,24,25], which seems to take place in parotid glands of psoriasis patients without salivation disorders (no changes in the studied nitrosative stress parameters in SWS). We observed excessive amount of NO and peroxynitrite in unstimulated and stimulated saliva of patients with hyposalivation. A positive correlation between TNF-α and NO concentrations in NWS of patients with hyposalivation and between IL-2 and NO content in NWS of patients with normal salivation confirm the previous observations that proinflammatory cytokines lead to the expression of *i*NOS in salivary gland cells, resulting in increased production of NO and its derivatives [46]. We also noted a boost in nitrosative stress (NO, S-nitrosothiols, peroxynitrite) and, primarily, nitrosative damage to protein elements of the salivary glands (S-nitrosothiols and nitrotyrosine) in unstimulated and stimulated saliva of psoriasis patients with hyposalivation vs. those with normal saliva secretion.

Evidence has shown that much larger amounts of NO generated in response to inflammation are connected with the cytotoxic effect of NO due to its interaction with superoxide anions to form peroxynitrite and other free radicals. Research results have revealed that intense production of NO and peroxynitrite in salivary glands acts as a strong proapoptotic agent [46,47]. Moreover, it has been observed that NO, by auto-ADP (adenosyno-diphospate) ribosylation of glyceraldehyde 3-phosphate dehydrogenase, inhibits the production of ATP that is necessary to maintain anabolic processes in the cell [23]. It has been demonstrated that apoptosis of salivary gland structures disturbs their function, and ATP deficiency impairs mechanisms responsible for replacing damaged or lost cellular elements [46,48]. We noted a negative correlation between NO and NWS secretion, and between peroxynitrite and protein concentrations in SWS of hyposalivation patients. These results suggest that decreased salivary secretion and protein synthesis/selection could be caused by the proapoptotic effect of NO on the salivary gland cells. This hypothesis requires further confirmation in histological studies. On the other hand, it is known that TNF-α and IL-2 stimulate the production of metalloproteinases, which results in structural changes in the basement membrane of the salivary glands [49]. A negative correlation between TNF-α and NWS secretion, and IL-2 and SWS secretion in patients with hyposalivation may result from damage to acinar cell-basement membrane interaction resulting from overproduction of MMPs (metalloproteinases) followed by decreased number of secretory units (acini and ducts) [50,51]. This phenomenon has been recently demonstrated in the saliva of Sjögren’s syndrome patients [52]. Remodeling of the extracellular matrix, alongside apoptosis, could be the reason for the observed drop in the synthesis/secretion of proteins and reduced salivary secretion in psoriasis patients with hyposalivation. It is noteworthy that salivary gland dysfunction occurs in patients with a longer duration and higher intensity of the disease.

The lack of significant differences in the secretion of NWS and SWS as well as proteins between patients with normal salivation (shorter disease duration, lower PASI) and the controls suggests that, at an early stage of the disease, the mechanisms of controlling saliva secretion and protein production/secretion counteract the damaging effects of psoriasis. Interestingly, already at this early stage we observed decreased amylase activity in SWS and UWS of patients with normal salivation, as well as intensification of this phenomenon in the saliva of patients with hyposalivation. These results may explain the negative correlation between peroxynitrite concentration and salivary amylase activity in NWS and SWS of patients with normal salivation. It has been demonstrated that peroxynitrite reacts readily with iron-sulfur cluster of several enzymes and is able to oxidize the sulfhydryl groups of proteins, leading to the formation of disulfides and resulting in their inactivation [53]. Naturally, it should be remembered that exposure to peroxynitrite entails tyrosine nitration of proteins [54]. This mechanism of amylase inactivation should be eliminated, as increased nitrotyrosine concentration was only observed in NWS and SWS of patients with hyposalivation, both compared to the controls and patients with normal saliva secretion.

Salivary glands are surrounded by a dense network of blood vessels that enable the exchange of components between the acinar cells and ducts, as well as blood. Thus, biomarkers present in the blood can permeate into the structures of salivary glands and hence into saliva. Therefore, saliva is more and more frequently considered a potential source of biological markers for systemic diseases. Many of the examined parameters clearly differentiated psoriatic patients with hyposalivation from psoriatic patients with normal saliva flow, and thus the levels of NO, nitrotyrosine, and IL-2 in NWS deserve special attention and should be further evaluated. Additionally, the observed positive correlation of PASI and TNF-α and IL-1β in UWS of patients with hyposalivation could provide a new non-invasive and simple method in the diagnosis of the intensity of the disease.

## 5. Conclusions

Increased levels of TNF-α, IL-2, and INF-γ, as well as decreased content of IL-10 in NWS and SWS of psoriasis patients compared to the controls indicated an imbalance between Th1 and Th2 cells in the salivary glands.

The severity of inflammation and nitrosative stress in the salivary glands of psoriatic patients depends on the disease duration.

At an early stage of the disease, the mechanisms controlling saliva secretion and protein production/secretion counteract the damaging effects of psoriasis. With the severity and duration of psoriasis, the secretory function of all salivary glands is lost, which is manifested as significant reduction of unstimulated and stimulated saliva secretion as well as protein concentration.

Dysfunction of salivary glands in the course of psoriasis may be attributed to inflammation and nitrosative stress.

## Figures and Tables

**Table 1:** Clinical characteristics of plaque psoriasis patients and control subjects.

Patient Characteristics		Control *n* = 60	PN *n* = 30	PH *n* = 30
**Sex**	Male *n* (%)	23 (38.33%)	13 (43.33%)	10 (33.33%)
	Female *n* (%)	37 (61.67%)	17 (56.67%)	20 (66.67%)
**Age (years)**		52 ± 5	49 ± 6	51 ± 3
**Height (cm)**		172 ± 6	176 ± 3	169 ± 8
**Weight (kg)** **Duration of psoriasis (years)**		75 ± 1	72 ± 3	74 ± 1
		ND	9.7 ± 3.4	18.52 ± 7.8
**PASI**		ND	10.39 ± 2.4	18.29 ± 4.6
**Psoriasis in the family**	<1 *n* (%)	ND	18 (60%)	6 (20%)
	≥1 *n* (%)	ND	12 (40%)	24 (80%)
**Blood Tests**				
**RBC (10^6^/µL)**		4.54 ± 0.54	4.23 ± 0.65	4.89 ± 0.87
**HCT (%)**		40.25 ± 6.5	42.45 ± 4.25	45.21 ± 0.89
**PLT (10^3^/µL)**		275 ± 56	245 ± 87	292 ± 23
**WBC (10^3^/µL)**		6.5 ± 2.2	7.05 ± 1.8	6.98 ± 1.86
**CRP (mg/L)**		1.5 ± 0.5	8.56 ± 6.3	6.32 ± 7.56
**Glc (mg/dL)**		69 ± 9.8	72 ± 9.8	75 ± 6.5
**ALT (U/L)**		27.56 ± 12.3	24.36 ± 9.68	27.24 ± 6.35
**AST (U/L)**		28.54 ± 12.25	30.23 ± 6.52	28.98 ± 8.64
**Dental Characteristics**				
**DMFT**		20 ± 3	19 ± 6	21 ± 2
**GI**		0.2 ± 0.1	0.2 ± 0.2	0.1 ± 0.2
**PPD**		1.5 ± 0.5	1.0 ± 0.5	1.0 ± 0.5
**Dental implants**		0	0	0

Abbreviations: ALT—alanine transferase; AST—aspartate transaminase; CRP—C-reactive protein; DMFT—decayed, missing, filled teeth index; GI—gingival index; Glc—D-glucose; HCT—hematocrit; *n*—number of patients; PASI—Psoriasis Area and Severity Index; PBI—papilla bleeding index; PLT—platelets; RBC—red blood cells; WBC—white blood cells. ND- not defined.

**Table 2:** ROC analysis of the assessed cytokines and nitrosative stress biomarkers in the saliva of plaque psoriasis patients with normal salivation and hyposalivation.

			NWS					SWS					
Parameter	AUC	95% Confidence Interval	*p*-Value	Cut-Off	Sensitivity (%)	Specificity (%)	AUC	95% Confidence Interval	*p*-Value	Cut-Off	Sensitivity (%)	Specificity (%)	AUC
**Cytokines**													
**TNF-α (pg/mg protein)**	0.68	0.5417–0.8183	**0.0166**	>15.83	63.33	63.33	0.7311	0.6025–0.8597	**0.0021**	>5.717	66.67	66.67	0.5611
**IL-2 (pg/mg protein)**	0.8111	0.7024–0.9198	**<0.0001**	>6.789	73.33	73.33	0.6478	0.5071–0.7884	**0.0493**	>1.595	63.33	63.33	0.6122
**IL-10 (pg/mg protein)**	0.7089	0.5760–0.8418	**0.0054**	<0.9704	63.33	63.33	0.7267	0.5997–0.8536	**0.0026**	<0.2410	66.67	66.67	0.6556
**INF-γ (pg/mg protein)**	0.6622	0.5246–0.7999	**0.0309**	>4.358	60.00	60.00	0.6278	0.4848–0.7708	0.0891	>1.142	60.00	60.00	0.5378
**Nitrosative Stress**													
**NO (ng/mg protein)**	0.77	0.6507–0.8893	**0.0003**	>318.90	66.67	66.67	0.6464	0.5443–0.7885	0.0556	>224.00	60.71	60.00	0.5244
**S–nitrosothiols (nM/mg protein)**	0.6844	0.5479–0.8209	**0.0141**	>42.73	60.00	60.00	0.7211	0.5857–0.8565	**0.0033**	>34.60	70.00	70.00	0.6644
**Peroxynitrite (nM/mg protein)**	0.7056	0.5744–0.8367	**0.0062**	>101.50	63.33	63.33	0.69	0.5569–0.8231	**0.0115**	>45.31	60.00	60.00	0.5744
**Nitrotyrosine (pM/mg protein)**	0.7444	0.6120–0.8769	**0.0011**	>3398.00	70.00	70.00	0.6611	0.5223–0.8000	**0.0321**	>2139.00	60.00	60.00	0.5011

Abbreviations: AUC—area under the curve; C—the control; IL-2—interleukin 2; IL-10—interleukin 10; INF-γ—interferon-gamma; NO—nitric oxide; NWS—non-stimulated whole saliva; SWS—stimulated whole saliva; TNF-α—tumor necrosis factor-alpha.

**Table 3:** ROC analysis of the assessed cytokines and nitrosative stress biomarkers in the plasma of plaque psoriasis patients with normal salivation and hyposalivation.

Plasma						
Parameter	AUC	95% Confidence Interval	*p*-Value	Cut-Off	Sensitivity (%)	Specificity (%)
**Cytokines**						
**TNF-α (pg/mL)**	0.5611	0.4135–0.7087	0.4161	>3.748	50.00	50.00
**IL-2 (pg/mL)**	0.6122	0.4685–0.7559	0.1354	>7.201	60.00	60.00
**IL-10 (pg/mL)**	0.6556	0.5127–0.7984	**0.0385**	>0.3863	63.33	63.33
**INF** **-γ (pg/mL)**	0.5378	0.3897–0.6858	0.6152	<251.20	50.00	50.00
**Nitrosative Stress**						
**NO (ng/mg protein)**	0.5244	0.3743–0.6746	0.745	>99.49	46.67	46.67
**S–nitrosothiols (nM/mg protein)**	0.6644	0.5261–0.8028	**0.0287**	>57.03	60.00	60.00
**Peroxynitrite (nM/mg protein)**	0.5744	0.4261–0.7228	0.3219	>302.90	60.00	60.00
**Nitrotyrosine (pM/mg protein)**	0.5011	0.3528–0.6494	0.9882	<3747.00	50.00	50.00

Abbreviations: AUC—area under the curve; C—the control; IL-2—interleukin 2; IL-10—interleukin 10; INF-γ—interferon-gamma; NO—nitric oxide; NWS—non-stimulated whole saliva; SWS—stimulated whole saliva; TNF-α—tumor necrosis factor-alpha.

**Table 4:** Statistically significant correlations in patients with plaque psoriasis.

Pair of Variables	Group	*r*	*p*
NO NWS and NWS flow rate	PH	−0.68	0.001
Peroxynitrite SWS and total protein SWS	PH	−0.56	0.0015
TNF-α NWS and NWS flow rate	PH	−0.60	0.004
IL-2 SWS and SWS flow rate	PH	−0.54	0.002
Peroxynitrite NWS and amylase NWS	PN	−0.58	0.0008
Peroxynitrite SWS and amylase SWS	PN	−0.68	<0.0001
TNF-α NWS and NO NWS	PH	0.60	0.004
IL-2 NWS and NO NWS	PN	0.64	0.002
TNF-α NWS and PASI	PH	0.59	0.0006
IL-2 NWS and PASI	PH	0.63	0.0029
Nitrotyrosine NWS and disease duration	PN	0.53	0.003
Nitrotyrosine SWS and disease duration	PN	0.58	0.001
Nitrotyrosine NWS and disease duration	PH	0.61	0.004
Nitrotyrosine SWS and disease duration	PH	0.60	<0.0001

Abbreviations: IL-2—interleukin 2; NO—nitric oxide; NWS—non-stimulated whole saliva; PN—psoriasis patients with normal salivation; PH—psoriasis patients with hyposalivation; SWS—stimulated whole saliva; TNF-α—tumor necrosis factor-alpha.
